# Activity of daily living in mucopolysaccharidosis IVA patients: Evaluation of therapeutic efficacy

**DOI:** 10.1002/mgg3.1806

**Published:** 2021-10-08

**Authors:** Hui Chen, Shaukat Khan, Betul Celik, Yasuyuki Suzuki, Yasuhiko Ago, Shunji Tomatsu

**Affiliations:** ^1^ University of Delaware Newark DE USA; ^2^ Nemours/Alfred I. duPont Hospital for Children Wilmington DE USA; ^3^ Medical Education Development Center Graduate School of Medicine Gifu University Gifu Japan; ^4^ Department of Pediatrics Graduate School of Medicine Gifu University Gifu Japan; ^5^ Department of Pediatrics Thomas Jefferson University Philadelphia PA USA

**Keywords:** activity of daily living, enzyme replacement therapy, hematopoietic stem cell transplantation, Morquio A, MPS IVA

## Abstract

**Background:**

Mucopolysaccharidosis IVA (MPS IVA, also called Morquio A syndrome) is caused by a deficiency of N‐acetylglucosamine‐6‐sulfate sulfatase (*GALNS*) and results in skeletal dysplasia symptoms such as short stature and abnormal gait. Treatments include enzyme replacement therapy (ERT) and hematopoietic stem cell transplantation (HSCT), but the effects are limited depending on the age of initiation and clinical phenotype. Thus, this study aims to assess the effects of treatments on MPS IVA patients compared to untreated MPS IVA patients and an age‐matched control group.

**Methods:**

We used activity of daily living (ADL) survey with 4 sections: “movement,” “movement with cognition,” “cognition,” and “other MPS symptoms.” Lower scores indicate more assistance required. This study included 161 patients, 270 total surveys, and 70 patients with longitudinal data.

**Results:**

We describe 134 severe patients and 25 attenuated patients. ERT and HSCT treatment improved only the “other MPS symptoms” section in severe patients. There were no differences between ERT and HSCT severe patient scores. A 19‐year‐old male patient, who had robust physical training, provided a significant increase in “movement” without treatment, suggesting the importance of exercise.

**Conclusion:**

Overall, this ADL questionnaire has demonstrated validation and reliability in assessing the MPS IVA patients and therapeutic efficacy.

## Graphical abstract

We have evaluated activity of daily living in mucopolysaccharidosis IVA patients from 161 patients. This is the first manuscript to evaluate therapeutic efficacy in both HSCT and ERT.

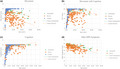

## 1 INTRODUCTION

Mucopolysaccharidoses (MPS) are a subset of lysosomal storage disorders (LSDs) that result in an accumulation of glycosaminoglycans (GAGs) (Meikle et al., 1999; Platt et al., 2018). The prevalence for MPS is about 1/25,000, with the most common types as MPS I and II, which occur in about 1/100,000 births (Federhen et al., 2020; Khan et al., 2017; Poorthuis et al., 1999). MPS IV (Phenotype MIM number, 253000) involves a deficiency in either the N‐acetylgalactosamine‐6‐sulfate sulfatase (*GALNS*; Gene MIM number, 612222; EC 3.1.6.4) enzyme for MPS IVA, also called Morquio A syndrome or β‐galactosidase for MPS IVB (*GLB1*; Gene MIM number, 611458) (Phenotype MIM number, 253010) (Khan et al., 2017). MPS IVA is the more common subtype, with a birth prevalence ranging from 1/71,000 to 1/179,000 (Giraldo et al., 2018; Leadley et al., 2014).

The GALNS enzyme deficiency results in GAGs accumulation, keratan sulfate (KS) and chondroitin‐6‐sulfate (C6S), in mainly bone, cartilage, heart valves, and cornea (Khan et al., 2017). The most common initial symptoms are short stature, abnormal gait with genu valgum, kyphosis, and pectus carinatum (Montaño et al., 2007). Other symptoms include respiratory and cardiac involvement but normal cognitive development, unlike other MPS types (Baujat & Valayannopoulos, 2014; Davison et al., 2013). Airway obstruction is a common, life‐threatening occurrence where the length of the trachea outgrows skeletal growth resulting in the twisted and narrowing trachea (Averill et al., 2021; Doherty et al., 2018; Pizarro et al., 2016; Tomatsu et al., 2016). Heart valve thickening and regurgitation, impaired diastolic filling, and higher heart rates progress as patients age (Kampmann et al., 2016; Leong et al., 2019). As about 75% of the cases present, severe phenotypes tend to exhibit initial symptoms before age of one year and tend to be diagnosed before 5 years (Montaño et al., 2007). Attenuated phenotypes progress more gradually as initial symptoms, and diagnosis is made after 5 years (Hendriksz et al., 2013; Tüysüz et al., 2019).

Clinical diagnosis of MPS IVA begins with the appearance or radiographic imaging of skeletal abnormalities (Wood et al., 2013). In severe phenotypes, symptoms, such as gibbus deformity, which involves kyphosis in the upper lumbar and lower thoracic vertebrae, can be observed at birth (Montano et al., 2008; Ohashi et al., 2009; Peracha et al., 2018; Sawamoto et al., 2020).

Total urine GAGs levels have low sensitivity and a high false‐positive rate in screening and diagnosing of MPS IVA patients (Ellsworth et al., 2016; Gray et al., 2007; Tomatsu et al., 2005; Whitley et al., 1989, 2002). KS and C6S levels in blood and urine are elevated by using ELISA or liquid chromatography‐tandem mass spectrometry (LC‐MS/MS) and correlate with clinical severity (Chin et al., 2020; Hintze et al.,; Shimada et al., 2014; Tomatsu et al., 2004; Wood et al., 2013). Notably, KS elevation is not exclusive to MPS IVA, but other MPS types can show the secondary elevation (Auray‐Blais et al., 2016; Ellsworth et al., 2016; Rowan et al., 2013; Tomatsu et al., 2005, 2014). Either fluorometric technique (Camelier et al., 2011; Ullal et al., 2014) or LC‐MS/MS (Scott et al., 2020) can demonstrate a deficiency of GALNS level. GALNS activity analysis using dried blood spots can be used in newborn screening, allowing early diagnosis (Chien et al., 2020; Stapleton et al., 2020). Thus, two‐tiered screening with the enzyme activity and GAG is useful for reducing false‐positive rates.

Interventions for MPS IVA address the combination of non‐skeletal and skeletal symptoms. Hip reconstruction, knee arthroplasty, and other surgical corrections can address skeletal deformities in the appendicular skeleton, such as hip dysplasia, genu valgum, ankle valgus, and abnormal gait (Celik et al., 2021; Montaño et al., 2007). In the spinal cord, craniocervical decompression with or without occipito‐cervical fusion can address spinal cord compression, stenosis, and instability (Broomfield et al., 2018; Krenzlin et al., 2018; Solanki et al., 2013). Adenoidectomy/tonsillectomy may address the airway obstruction due to narrowing caused by enlarged GAG deposits (Berger et al., 2013). Tracheal narrowing, commonly resulting in a decreased space at the thoracic inlet, is evident as early as 2 years old and progresses over time (Averill et al., 2021; Tomatsu et al., 2016). An autopsied case of severe MPS IVA showed that the patient died at age 23 of tracheal obstruction and respiratory failure due to cord myelopathy (Doherty et al., 2018). Another 16‐year‐old male case receiving ERT for 2.5 years with severe tracheal narrowing recovered from respiratory failure by tracheal reconstructive surgery (Pizarro et al., 2016). Tracheal and vascular reconstruction relieved tracheal narrowing by resection and anastomosis and horizontal course of the innominate artery trunk compressing the trachea (Tomatsu et al., 2016). Less invasive management includes applying continuous positive airway pressure using a mask to facilitate oxygenation during sleep apnea (Berger et al., 2013). Valve replacements can address heart valve thickening, but cardiovascular involvements are typically minor (Nicolini et al., 2008). Keratoplasty addresses corneal clouding as it progresses over time, but rejection episodes can occur (Ohden et al., 2017).

There are several treatment options for MPS IVA. Enzyme replacement therapy (ERT), elosulfase alfa (trade name Vimizim), was approved by the United States Food and Drug Administration in 2014 (Drug Approval Package: Vimizim (Elosulfase Alfa), 2014). Elosulfase alfa doses at 2 mg/kg weekly normalized urinary KS levels, improved endurance using the 6‐minute walk test but not on the 3‐minute stair climb test, and stabilized pulmonary function using forced vital capacity and forced expiratory volume in 1 second over approximately 5 years (Cleary et al., 2021; Hendriksz, Burton, et al., 2014; Hendriksz et al., 2018). However, another long‐term study over 9 years found that pulmonary function still decreased when corrected for age, sex, and height (Wysong et al., 2012). As measured using left ventricular ejection fraction, cardiovascular functioning did not change in one study with a mean follow‐up of 4.9 years (Cleary et al., 2021). Cardiac hypertrophy stabilized and improved more in younger patients, but valvular heart disease only stabilized over 3–6 years on ERT (Lin et al., 2018). ERT did not improve bone growth despite initiating treatment before 5 years old with over 2 years of follow‐up (Doherty et al., 2019). Half of the patients who started ERT under 5 years old still stopped growing between 5 and 9 years of age, and ultimately, patients receiving ERT under 5 years old were of average MPS IVA height for their sex (Doherty et al., 2019). Although ERT benefits organomegaly, urine KS levels, and endurance, the effects on other symptoms such as the skeletal, cardiovascular, and pulmonary systems are minor. Blood KS did not decrease in parallel with urine KS reduction, suggesting that urine KS does not correlate with skeletal dysplasia improvement and that urine KS is not a suitable biomarker to measure therapeutic efficacy, only a pharmacokinetic biomarker (Fujitsuka et al., 2019).

Allogenic hematopoietic stem cell transplantation (HSCT) provides the deficient enzyme (Nowak, 2008; Taylor et al., 2019; Wang et al., 2016). In a long‐term study of over 10 years post‐transplantation, all 4 MPS IVA patients demonstrated the maintenance of GALNS activity at donor levels, delay in skeletal dysmorphic progression without orthopedic surgery in an ambulant condition (Yabe et al., 2016). However, there is still only a minor impact on growth even if HSCT started at 4 years of age (Yabe et al., 2016). The patient with MPS IVA demonstrated significant improvements in airway obstruction and hypermobile joints, minor improvements in height, and stabilization of spinal cord compression 1‐year post‐transplantation (Wang et al., 2016). The benefits of HSCT are that it is a one‐time treatment that can maintain normal enzyme levels over the long term compared to ERT, a weekly infusion. This makes ERT less cost‐effective. HSCT also can target the bone and cartilage more effectively than ERT because of GALNS expression in the bone marrow.

The activity of daily living (ADL) is one form of the patient (family) ‐reported outcome that evaluates the patient's ability to function independently in routine daily life. MPS IVA has systemic symptoms that can lead to poor endurance, pain, fatigue, and mobility restrictions from skeletal dysplasia. ADL questionnaires can detect more subtle improvements in movement, pain, and general quality of life when clinical improvements are not significant. Not all clinical trials include this measurement as an outcome, and when it is included, the questionnaires are not standardized. Several questionnaires are used for ADL assessment.

A natural history study (MorCAP) with 325 untreated MPS IVA patients were evaluated for up to 10 years starting in 2008. Untreated patients demonstrated impairment in endurance correlating with urinary KS levels and increased use of pain medication with age (Harmatz et al., 2013). This natural history program uses the health assessment questionnaire, containing 52 questions in 10 categories – dressing, bathing, eating/drinking, toothbrushing, toileting, grooming, mobility, walking, stairs, and caregiver assistance (Harmatz et al., 2013). There are 11 symptom questionnaires, 9 functional capacity questionnaires, 7 health‐related questionnaires, and 2 questionnaires addressing the impact on family and caregivers (Hendriksz et al., 2016). Most common questionnaires for MPS IVA include the health assessment questionnaire and the adolescent, pediatric pain tool (Hendriksz et al., 2016). Unlike other questionnaires such as the Functional Independence Measure (Guarany et al., 2012), our ADL questionnaire does not need a trained professional to administer, which makes sending, completing, and returning the questionnaire more convenient for the patient and their families (Yasuda et al., 2016). The ADL survey used has 4 sections: “movement,” “movement with cognition,” “cognition,” and “other MPS symptoms.” The “movement” section evaluates walking, stairs, hand movements, and endurance. The “movement with cognition” section evaluates toileting, changing clothes, bathing, and eating. The “cognition” section evaluates understanding, conversation, social participation, and problem‐solving. The “other MPS symptoms” section evaluates work/study, behavioral problems, sleep, pain, joint flection, respiratory status, infection, vision, hearing, skin, hair, and appetite. Our questionnaire is different from other questionnaires in that our survey includes a section specific to MPS symptoms which is not evaluated in other questionnaires. It also evaluates cognition which is not covered in the health assessment questionnaire.

ERT has shown some improvements in patient‐reported outcomes, but not consistently. Higher dependence on a wheelchair correlated with decreased scores in the mobility, self‐care, and usual activities section of the EuroQol 5 dimensions, 5 levels score‐which measures mobility, self‐care, usual activities, pain, and anxiety/depression (Hendriksz, Lavery, et al., 2014). Compared to untreated MPS IVA patients, ERT significantly improved mobility and self‐care scores in the health assessment questionnaire but had no impact on the caregiver assistance section for 63 adult patients (18 years old or older) over 2 years (Hughes et al., 2017). Another clinical trial over 2 years confirmed these findings in 169 patients with an average age of 14.4 ± 10.3 years old (range 5–57.4 years old) and added that caregiver assistance scores improved if above the mean at baseline, but not below the mean (Hendriksz et al., 2018). ERT treatment improved scores in the health assessment questionnaire, specifically the self‐care and mobility sections, of 51 patients with an average age of 14.9 years (range 2–58 years) over 3 years. In the same study, pain severity remained stable over time, and wheelchair status remained unchanged (Cleary et al., 2021).

HSCT MPS IVA patients also increased patient‐reported outcomes, but the small sample sizes prevent statistical significance conclusions. Average ADL scores for walking, stairs, hand movements, endurance, toileting, changing clothes, bathing, and eating were increased in HSCT MPS IVA patients than untreated MPS IVA patients (Yabe et al., 2016). A case study suggested numerical improvements after 9 years post‐transplantation in the MPS specific symptoms section: work/study, sleep, joint pain, and respiratory infections (Chinen et al., 2014). Yasuda et al. compared the effect of ERT and HSCT on ADL scores with age‐matched control and untreated patients, but limitations included small sample sizes of only 82 total patients and 14 patients with longitudinal data (Yasuda et al., 2016).

This study uses the same ADL questionnaire as Yasuda et al. and others (Chinen et al., 2014; Lin et al., 2018; Tanjuakio et al., 2015; Yabe et al., 2016). The current study includes the largest number of subjects evaluated with the ADL questionnaire, including 161 patients, 270 total surveys, and 70 patients with longitudinal data. The earliest survey was collected in December 2012, and the newest survey was collected on March 24, 2021. This study aims to evaluate the effects of treatments on MPS IVA patients of different ages and phenotypes, compared to untreated MPS IVA patients and an age‐matched healthy control group.

## 2 MATERIALS AND METHODS

The study was conducted according to the guidelines of the Declaration of Helsinki and approved by the Institutional Review Board of Nemours/AIDHC (IRB #750932: 06/05/2015, initial approval: IRB # 750932; 02/10/2021, Amendment/Modification approval). MPS IVA patients and healthy controls were enrolled and informed with consent at Alfred I. duPont Hospital for Children, Wilmington, Delaware (USA) and Gifu University (Japan). Informed consent was also obtained from parents and caregivers. There are 145 healthy controls with an average age of 9.19 years, range 0.33 to 43.55 years of age. Healthy control data are the same as those used in previously published ADL data of MPS IVA and MPS II (Montano et al., 2008; Yasuda et al., 2016). Age, height, weight, treatment history, surgical history, sex, and ADL and MPS questionnaires were collected. A total of 161 MPS IVA patients were enrolled, 74 females and 87 males, with 270 surveys. Severe phenotypes were determined by having a final height below the 75th percentile of the established MPS IVA growth curve accounting for each sex (Montano et al., 2008; Tomatsu et al., 2012). One hundred thirty‐four patients expressed the severe phenotype, and 25 expressed the attenuated phenotype. Two patients had an unknown phenotype because the height was not available. Heights were measured in the standing position, or if needed, in a supine position with knees flattened and legs fully extended. The body mass index (BMI) was calculated by dividing the weight in kilograms by the height in meters squared. Eighty‐two patients had a history of treatment with either ERT or HSCT. ERT involved weekly infusions of elosulfase alfa (2.0 mg/kg) for 1 year or longer. Seventy‐four patients had ERT treatment, and 8 patients had HSCT. Seventy‐nine patients did not have any of the listed treatments.

Seventy patients had multiple surveys for longitudinal data; 50 of these patients had a history of treatment with either ERT or a HSCT, and 20 of these patients with longitudinal data did not have any of the listed treatments.

The ADL survey used had 4 sections as validated and described in healthy controls previously (Chinen et al., 2014; Tanjuakio et al., 2015; Yabe et al., 2016; Yasuda et al., 2016): “movement,” “movement with cognition,” “cognition,” and “other MPS symptoms.” Changes in “movement,” “cognition,” “movement with cognition,” and “other MPS symptoms” scores were determined using the first survey as the baseline.

The “movement,” “movement with cognition,” “cognition” sections were on a scale of 0–5, with higher scores indicating healthy and independent functioning. The maximum score for the “movement,” “movement with cognition,” “cognition” sections was 20, while the maximum score for the “other MPS symptoms” section was 60. The “movement” section evaluated walking, stairs, hand movement, and endurance. The “movement with cognition” section evaluated toileting, changing clothes, bathing, and eating. The “cognition” section evaluated understanding, conversation, social participation, and problem‐solving. The “other MPS symptoms” section evaluated work/study, behavioral problems, sleep, pain, joint flection, respiratory status, infections, vision, hearing, skin, hair, and appetite.

**FIGURE 1:**
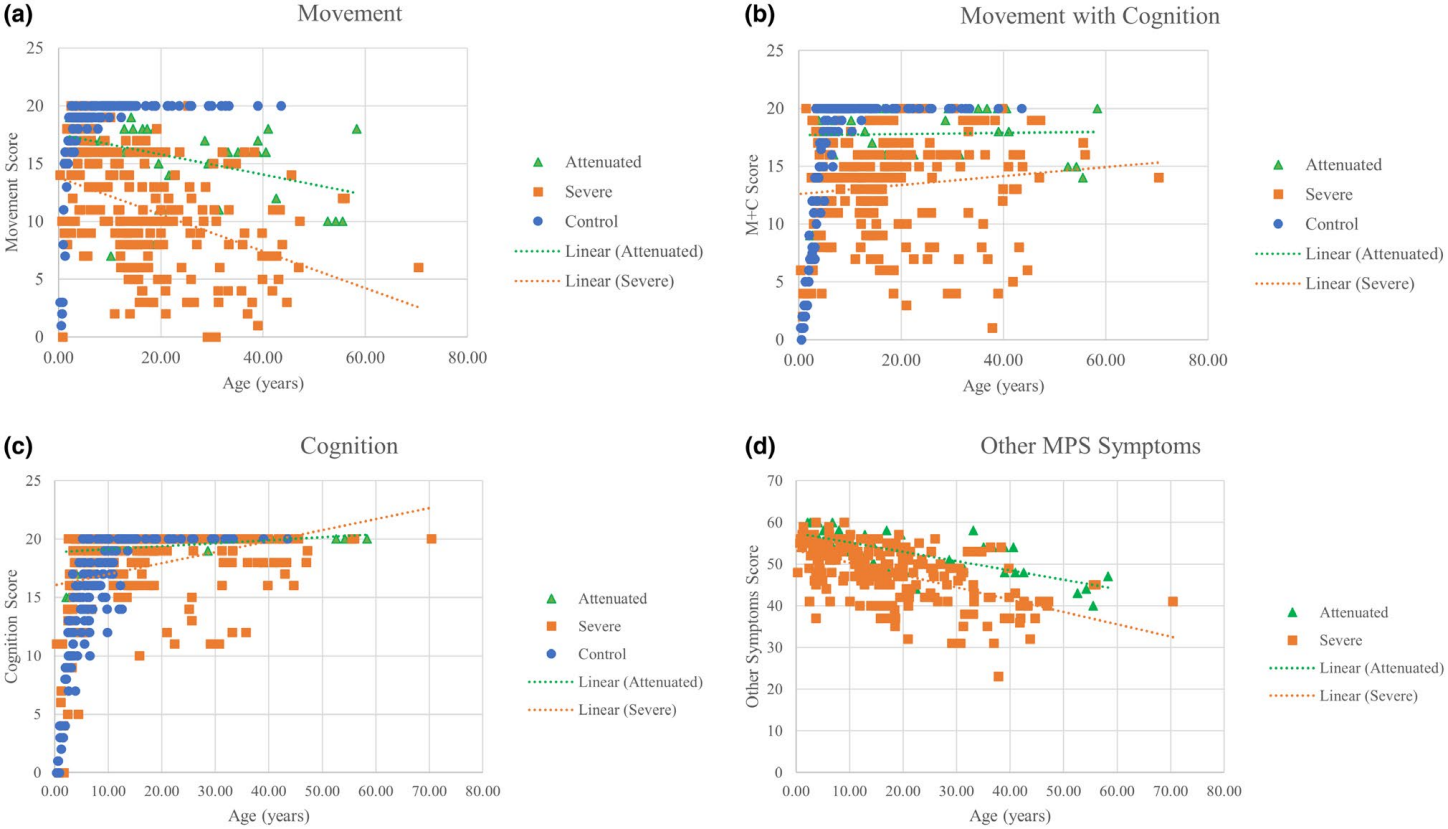
Age‐dependent ADL scores for each MPS IVA patient according to phenotype and age‐matched controls. (a) “Movement” scores. Attenuated trend line: *y* = −0.0873*x* + 17.54, *R* ^2^ = 0.180. Severe trendline: *y* = −0.1592*x* + 13.798, *R* ^2^ = 0.160. (b) “Movement with cognition” scores. Attenuated trend line: *y* = 0.0047*x* + 17.69, *R* ^2^ = 0.0007. Severe trendline: *y* = 0.0388*x* + 12.608, *R* ^2^ = 0.0116. (c) “Cognition” scores. Attenuated trend line: *y* = 0.0259*x* + 18.867, *R* ^2^ = 0.0973. Severe trendline: *y* = 0.0943*x* + 16.063, *R* ^2^ = 0.0969 (d) “Other MPS symptoms” score. Attenuated trend line: *y* = −0.2234*x* + 57.393, *R* ^2^ = 0.449. Severe trendline: *y* = −0.2952*x* + 53.274, *R* ^2^ = 0.311

Descriptive statistics were used for Tables 1 and 2. *p*‐values for all t‐tests conducted were two‐tailed unequal variances. T‐tests were used for Tables 1 and 2. Significance was determined by a *p*‐value smaller than 0.05. Standard deviations were assumed as a sample of the population. Outliers were determined by being larger than 150% of the interquartile range above the 3rd quartile or below the 1st quartile. This outlier boundary is commonly used in a boxplot to visualize distributions. ANOVA single factor was used for Table 2, with an alpha of 0.05. Linear regression was used for Figures 1 and 3.

**TABLE 1:** Demographics in MPS IVA patients

		Control	All MPS IVA	Severe MPS IVA	Attenuated MPS IVA
N		145	161	134	25
Males: females, n		72:73	87:74	73:61	13:12
Average final age (years) ± SD		9.19 ± 8.00	18.84 ± 14.12	18.24 ± 13.78	22.38 ± 16.06
	Final age range (years)	0.33–43.55	0.33 to 70.41	0.33 to 70.41	3.89 to 58.33
Average final BMI (kg/m^2^) ± SD		N/A	22.87 ± 6.72	23.19 ± 7.00	21.20 ± 4.74
Patients received surgeries, n (%)		0 (0)	83 (51.6)	69 (51.5)	13 (52.0)
	Hip, n (%)		24 (14.9)	17 (12.7)	7 (28.0)
	Spine, n (%)		34 (21.1)	31 (23.1)	2 (8.0)
	Leg, n (%)		45 (28)	40 (29.9)	4 (16.0)
	Adenoids and tonsils, n (%)		14 (8.7)	11 (8.2)	3 (12.0)
	Ears, n (%)		13 (8.1)	11 (8.2)	1 (4.0)
	Trachea, n (%)		10 (6.2)	10 (7.46)	0 (0)
	Inguinal and umbilical hernias, n (%)		4 (2.5)	4 (3.0)	0 (0)
	Other, n (%)		8 (5.0)	5 (3.7)	3 (12.0)

**TABLE 2:** ADL scores in controls and patients with severe phenotypes or attenuated phenotypes

Age range (years)					
		0 to 5	5 to 10	10 to 15	>15
Control	N	46	54	25	20
	Movement	14.96 ± 6.10	19.43 ± 0.57	19.96 ± 0.20	20.00 ± 0
	Movement with Cognition	9.46 ± 6.71	19.39 ± 1.47	19.88 ± 0.44	20.00 ± 0
	Cognition	8.87 ± 5.52	16.07 ± 2.50	18.76 ± 1.85	20.00 ± 0
Severe	N	37	33	40	115
	Movement	14.49 ± 4.84	14.94 ± 3.30***	9.95 ± 3.30***	9.12 ± 4.56***
	Movement with Cognition	10.95 ± 5.64	14.64 ± 3.44***	12.98 ± 3.06***	13.77 ± 4.63***
	Cognition	13.00 ± 6.08*	18.06 ± 2.54***	19.03 ± 1.64	18.68 ± 2.35***
	Other MPS Symptoms	52.32 ± 4.97	53.03 ± 4.05	49.40 ± 4.19	44.87 ± 6.73
Attenuated	N	5	7	6	25
	Movement	19.00 ± 0***	17.43 ± 1.40*	15.67 ± 4.41	14.40 ± 3.11***
	Movement with Cognition	14.20 ± 3.21	19.29 ± 1.50	18.67 ± 1.21	17.88 ± 2.15***
	Cognition	16.60 ± 2.08***	19.71 ± 0.76***	20.00 ± 0*	19.80 ± 0.58
	Other MPS Symptoms	59.40 ± 1.73	56.29 ± 3.09	52.83 ± 2.79	49.88 ± 4.94
t‐tests between phenotypes	Movement	*p* < 0.001	*p* = 0.0041	*p* = 0.0227	*p* < 0.001
	Movement with Cognition	*p* = 0.0454	*p* < 0.001	*p* < 0.001	*p* < 0.001
	Cognition	*p* = 0.0268	*p* = 0.035	*p* = 0.006	*p* < 0.001
	Other MPS Symptoms	*p* = 0.0028	*p* = 0.0365	*p* = 0.0288	*p* = 0.001

Note

ANOVA *p*‐values: Severe groups 0 to 5, 5 to 10, 10 to 15, >15: “movement,” “cognition,” and “other MPS symptoms” *p*‐value < 0.001; “movement with cognition” *p*‐value = 0.002. Attenuated groups 0 to 5, 5 to 10, 10 to 15, >15: “movement” *p*‐value 0.0086, “movement with cognition” *p*‐value 0.0079, “cognition” and “other MPS symptoms” *p*‐value < 0.001.

t‐test *p*‐values:

* *p* < 0.05 of severe or attenuated phenotype scores compared to control.

** *p* < 0.01 of severe or attenuated phenotype scores compared to control.

*** *p* < 0.001 of severe or attenuated phenotype scores compared to control.

## 3 RESULTS

### 3.1 MPS IVA patient demographics

A total of 161 MPS IVA patients were enrolled. Twenty‐five patients were attenuated, and 134 patients were severe. Two patients had an unknown height, so that the phenotype could not be determined. The distribution between males and females was relatively even with slightly more males (Table 1). The average age of the last survey for attenuated patients was 22.38 ± 16.06 years (range 3.89 to 58.33 years), while the average age of the last survey for severe patients was 18.24 ± 13.78 years (range 0.33 to 70.41 years). The *p*‐value for the t‐test comparing the average final age of attenuated and severe patients was 0.236. The final average BMI for attenuated patients was 21.20 ± 4.74 kg/m^2^ and for severe patients was 23.19 ± 7.00 kg/m^2^, which were both healthy BMIs for an adult. Approximately half of the patients received any type of surgery, with leg surgeries—including the knee, tibia/fibula, femur, and ankle–being the most common surgery at 28%. Other surgeries included C‐sections and surgeries on the ovaries, jaw, hand, aorta, appendix, eye, and breast. Patients with the severe phenotype had a higher proportion of patients with leg (29.9% vs. 16%), spine (23.1% vs. 8%), ear (8.2% vs. 4%), and tracheal (7.46% vs. 0%) surgeries, whereas attenuated patients had a higher proportion of hip (28% vs. 12.7%) surgeries. The average age of patients with at least one surgical intervention was 21.9 ± 14.1 years old.

Eight patients were deceased (patient numbers 17, 28, 41, 46, 53, 81, 141, and 166) after ADL questionnaires were collected. Patients 17 (female) and 28 (male), sibling cases, died of the respiratory infection at 19 and 21 years old, respectively, during the COVID‐19 pandemic and stood at 88.8 cm and 92.8 cm tall. Both patients had short stature, sleep apnea, a wheelchair, and a narrowing trachea. Patient 41 (male), 92 cm tall, developed characteristic skeletal features by the age of 1.5 years and was wheelchair‐bound. He had severe narrowing of the trachea with a look‐up‐to‐the‐sky position. Five days later, the patient died of acute respiratory distress syndrome after occipito‐C1–C2 cervical fusion at 20 years old (Yasuda et al., 2013, 2016). Patient 46 (female), 84 cm tall, used a wheelchair with difficult breathing and died of respiratory failure after a common cold at 28 years old. Patient 53 (male), 100 cm tall, underwent tracheostomy and had continuous positive airway pressure (CPAP) at 17 years old. He died of respiratory failure at 37 years old. Patient 81 (male) developed characteristic skeletal abnormalities at the age of 4 months. By 5 years of age, the patient stopped growing, with his maximal height reaching 86.4 cm. The patient died of severe tracheal obstruction and hypoventilation originating from respiratory muscle weakness from neurological cord deficit due to cord myelopathy at 23 years (Doherty et al., 2018). Patients 141 (male) and 166 (male) were siblings and wheelchair‐bound at 91.4 and 90 cm tall. Both patients underwent 4 years of ERT. Both had sleep apnea and had bilevel positive airway pressure (BiPAP) with a narrowing trachea, suggesting the requirement of tracheal reconstructive surgery. Both patients died of sudden respiratory failure during physical activity at 25 and 26 years old. Overall, all deceased patients had restrictive and obstructive lungs with marked short stature (below the 25th percentile of Morquio growth chart). Patient numbers 17, 28, 46, 81, 141, and 166 were between the 3rd and 10th percentile, while patient numbers 41 and 53 were between the 10th and 25th percentile.

### 3.2 Control subjects

There were 145 control subjects, and over 60% were under 10 years old (Tables 1 and 2). These were the same control subjects used in Tanjuakio et al. (Tanjuakio et al., 2015). ADL scores increased in all 3 sections with age. Most “movement” and “movement with cognition” scores plateaued at the maximum score of 20 before age 10 (Figure 1). “Cognition” scores plateaued later at around age 12 (Figure 1). Healthy control subjects showed the plateau at the highest score for all movement‐based sections before age 10 and for “cognition” around age 11 (Yasuda et al., 2016).

All subjects had a maximum score of 20 in all sections by age 15.

### 3.3 ADL scores in severe and attenuated patients

There was a total of 225 surveys of MPS IVA patients with severe phenotype. “Movement with cognition” and “cognition” scores all increased with age, but “movement” and “other MPS symptoms” scores decreased with age (Table 2; Figure 1). “Movement with cognition” and “cognition” scores were significantly lower than age‐matched control subjects’ scores (Table 2). “Cognition” scores were lower for severe MPS IVA patients in all ages except for 10–15. In severe patients, all of the age groups were significantly different for each section using an ANOVA. The “movement,” “cognition,” and “other MPS symptoms” sections had *p*‐values less than 0.001. The “movement with cognition” had a *p*‐value of 0.002.

There was a total of 43 surveys of MPS IVA patients with the attenuated phenotype. Most of the surveys fell under the age group above 15 years old, so that the younger age groups may have had a lower accuracy from a small sample size. “Movement with cognition” and “cognition” scores increased with age, but some patients did not reach the maximum score (Table 2; Figure 1). Most “cognition” scores increased to the maximum score. When comparing the standard deviations for ages 5 and older, “cognition” scores have lower variation than other sections (Table 2). “Movement” and “other MPS symptoms” scores declined with age. Attenuated MPS IVA patients remained significantly lower in “movement” and “movement with cognition” scores than age‐matched controls even after age 15 (Table 2). “Cognition” scores started higher than age‐matched controls but reached a final score equivalent to control after age 15 (Table 2).

In all age groups, attenuated patients had higher scores in all categories surveyed, including “other MPS symptoms” than severe patients (Table 2). Using an ANOVA, all age groups were significantly different in each section. The *p*‐value for “movement” was 0.0086, and the *p*‐value for “movement with cognition” was 0.0079. The *p*‐values for “cognition” and “other MPS symptoms” were less than 0.001.

### 3.4 ADL scores for patients with ERT

There were 135 surveys in 82 patients with ERT (112 severe and 23 attenuated surveys). The average age for 61 severe patients under ERT was 15.84 ± 9.34 years, which was lower than the average age for 13 attenuated patients under ERT, which was 25.18 ± 16.41 years (*p*‐value = 0.014). Severe patients had a more extended treatment duration (3.46 ± 1.72 years) than attenuated patients (2.80 ± 1.33 years) with a *p*‐value of 0.047.

All severe ERT patients had a lower “movement” and “movement with cognition” score compared to control (Table S1). “Cognition” scores were higher for severe ERT patients than control, but this was likely due to the younger average age of control patients (Table 1). All attenuated ERT patients had no significant differences in section scores and control.

When compared to severe untreated patients, only “other MPS symptoms” were significantly increased (Table S1). All other sections for severe ERT patients were numerically higher but insignificant compared to severe untreated patients (Table S1). For attenuated patients, only the “movement” section was significantly increased with ERT treatment compared to untreated patients (Table S1). None of the other sections were significantly different compared to attenuated untreated patients.

### 3.5 ADL scores for patients with HSCT

There were 8 patients with HSCT, 7 severe (patient numbers 26, 69, 70, 101, 134, 145, 172) and 1 attenuated (patient number 71) phenotype. The average age for the 7 severe patients was 13.93 ± 14.61 years (range 1.73 to 25.3 years old). Severe HSCT and ERT patients have comparable average ages (*p*‐value = 0.744). The attenuated patient was 28.61 years old. The severe patients’ surveys were taken 0.192 to 11.2 years after HSCT with a mean of 2.90 ± 4.68 years, but 3 of 8 patients, including the attenuated patient, did not have a duration of HSCT available.

Patient number 26 (male) was 26 years old and 103 cm tall. Patient number 69 (female) was 25.3 years old and 113 cm tall. Patient number 70 (male) was 36 years old and 115 cm tall. Patient 71 (male) was 28.6 years old and 137 cm tall. Patient number 101 (female) was 2.5 years old and 81 cm tall. Patient number 134 (male) was 3.5 years old and 87 cm tall. Patient number 145 (female) was 2.5 years old and 86 cm tall. Patient 172 (male) was 1.73 years old and 86 cm tall.

When compared to control, severe HSCT patients had lower “movement” and “movement with cognition” scores (Table S1). “Cognition” scores were equivalent to control. Severe HSCT patients also had a higher score for “other MPS symptoms” than severe untreated patients, but none of the other sections were significantly different (Table S1).

**FIGURE 2:**
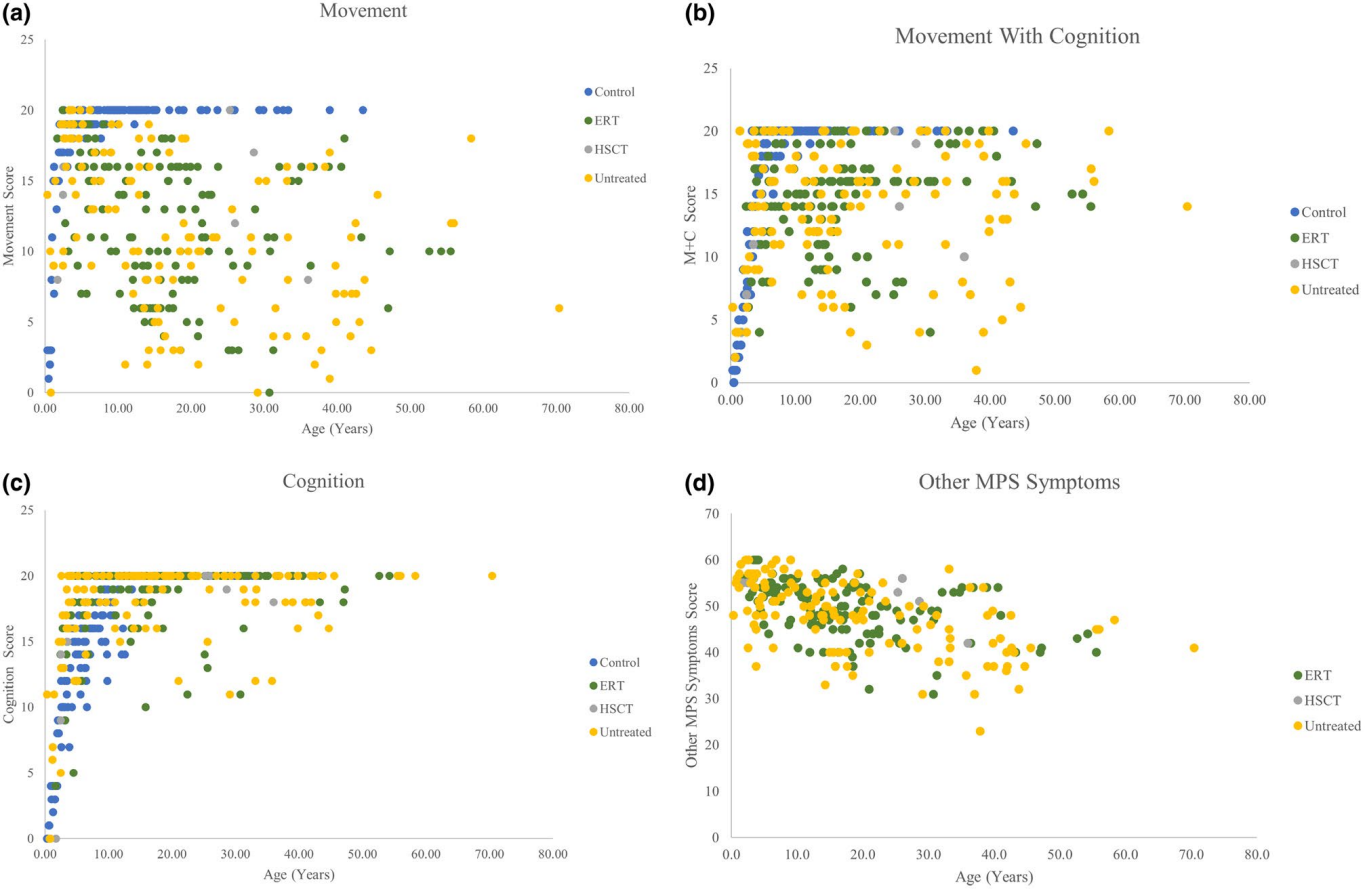
ADL scores compared with treatment groups. (a) Scores in “Movement.” (b) Scores in “Movement with cognition.” (c) Scores in “Cognition.” (d) Scores in “Other MPS symptoms scores.” ERT trendline: *y* = −0.2227*x* + 53.202. *R* ^2^ = 0.1856. Untreated trendline: *y* = −0.2735*x* + 53.153. *R* ^2^ = 0.2967. HSCT trendline: *y* = −0.2116*x* + 55.961. *R* ^2^ = 0.4511. Overall trendline: *y* = −0.2568*x* + 53.433. *R* ^2^ = 0.2567.

Severe HSCT and ERT patients did not have significantly different scores in any section (Table S1). Severe HSCT patients had a numerically higher “other MPS symptoms” score (52.86 ± 4.88) compared to severe ERT patients (48.88 ± 5.79), which was close to a *p*‐value less than 0.05 but not significant (*p*‐value = 0.0772) (Table S1). The HSCT trendline for “other MPS symptoms” had the strongest negative correlation with R2 = 0.4511 compared to ERT and untreated trendlines (Figure 2).

**FIGURE 3:**
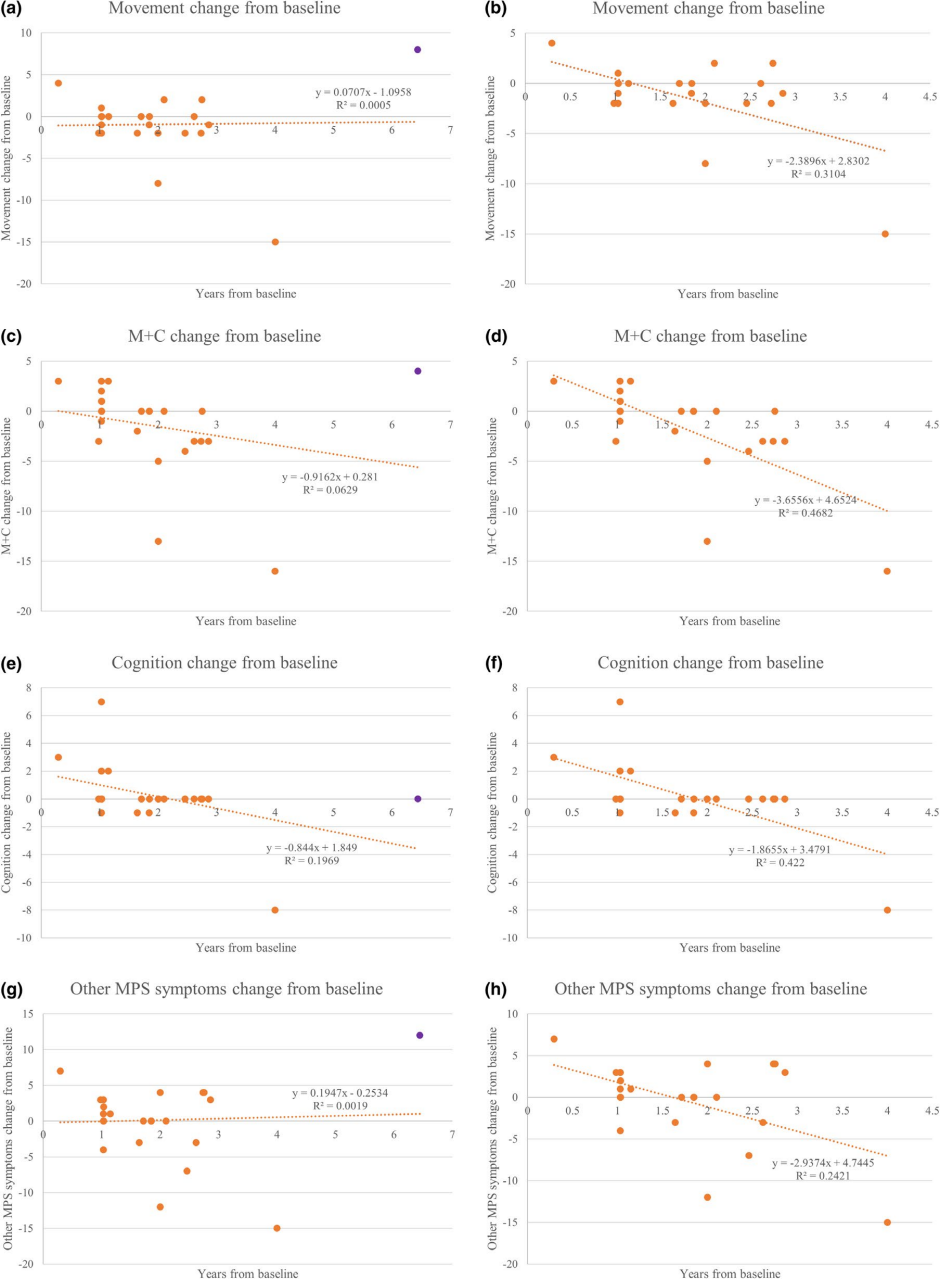
(a) “Movement” score changes over years from baseline for untreated severe patients. All surveys included. Linear regression line *y* = 0.0707*x*–1.0958. *R* ^2^ = 0.0005. (b) “Movement” score changes over years from baseline for untreated severe patients. Patient number 18 outlier (purple) excluded. Linear regression line *y* = −2.3896*x* + 2.8302. *R* ^2^ = 0.3104. (c) Movement with cognition” (M+C) score changes over years from baseline for untreated severe patients. All surveys included. Linear regression line *y* = −0.9162*x* + 0.281. *R* ^2^ = 0.0629. (d) Movement with cognition” (M+C) score changes over years from baseline for untreated severe patients. Patient number 18 outlier (purple) excluded. Linear regression line *y* = −3.6556*x* + 4.6524. R^2^ = 0.4682. (e) “Cognition” score changes over years from baseline for untreated severe patients. All surveys included. Linear regression line *y* = −0.844*x* + 1.849. *R* ^2^ = 0.1969. (f) “Cognition” score changes over years from baseline for untreated severe patients. Patient number 18 outlier (purple) excluded. Linear regression line *y* = −1.8655*x* + 3.4791. *R* ^2^ = 0.422. (g) “Other MPS symptoms” score changes over years from baseline for untreated severe patients. All surveys included. Linear regression line *y* = 0.1947*x* + 0.2534. *R* ^2^ = 0.0019. (h) Patient number 18 outlier (purple) excluded. Linear regression line *y* = −2.9374*x* + 4.7445. *R* ^2^ = 0.2421

### 3.6 ADL scores for untreated patients

There were 105 surveys from severe untreated patients and 19 surveys from attenuated untreated patients. Sixty‐six severe patients never had treatment. Eleven severe patients had ERT, but the duration was under one year at the survey time. Two severe patients had surveys obtained pre‐HSCT. The average age for severe patients was 19.60 ± 15.12 years, ranging from 70.4 to 0.33 years of age. Severe untreated patients were older than severe ERT (*p*‐value = 0.0299) but not severe HSCT patients (*p*‐value = 0.355). Twelve attenuated patients never had treatment. Five attenuated patients had pre‐ERT surveys, or the duration was not one year when the survey was taken. Attenuated patients had an average age of 18.16 ± 15.50 years with a range of 2.15 to 58.33 years of age. Attenuated ERT patients and untreated patients had comparable average ages (*p*‐value = 0.162).

All of the severe untreated patients had scored lower than those of control for “movement” and “movement with cognition” scores (Table S1). “Cognition” scores were higher for severe untreated patients than control, but this was likely due to the younger average age of control patients (Table 1). Attenuated patients had a lower “movement” score than control, but the average “cognition” score was higher than control, likely due to an older average age compared to control (Table S1). The “movement with cognition” score was not significantly different between attenuated untreated patients and controls.

Overall, treated and untreated patients had overlap in all of their section scores (Figure 2). With age, “movement” and “movement with cognition” scores had shown the most overt decline in treated patients than “cognition” scores (Figure 2). “Cognition” was less variable with age, and most patients reached the maximum score of 20 regardless of treatment (Figure 2). However, in the “other MPS symptoms” category, scores declined over time, regardless of treatment, as seen in the negative trendline slopes (Figure 2).

In attenuated patients, the “movement” and “cognition” sections were significantly different (*p*‐value < 0.001) when comparing the control, untreated, ERT, and HSCT groups using an ANOVA. For the “movement with cognition” section, the *p*‐value was 0.55, so there is no difference between the control, untreated, and any treatment. When comparing the treatment groups (ERT and HSCT) and untreated, the *p*‐values were all larger than 0.1. The “movement” section *p*‐value was 0.083 for the ERT, HSCT, and untreated groups.

In severe patients, all sections were significantly different when comparing the control, untreated, ERT, and HSCT groups using an ANOVA (*p*‐value < 0.001). When comparing the treatment groups ERT, HSCT, and untreated, the “cognition” and “other MPS symptoms” sections had a *p*‐value < 0.05. The “movement” section had a *p*‐value of 0.18, and the “movement with cognition” section had a *p*‐value of 0.078.

### 3.7 Change in ADL scores by treatment

A total of 50 treated patients and 20 untreated patients had multiple surveys available for longitudinal analysis. Two of the patients were treated with HSCT, and the other 48 patients were treated with ERT. There were no untreated attenuated patients who had multiple surveys.

For severe ERT patients, there was no difference between “movement,” “cognition,” and “other MPS symptoms” scores compared to untreated patients (Table S1). Severe ERT patients had a higher increase in “movement with cognition” than severe untreated patients (*p*‐value = 0.032).

When comparing the ERT, HSCT, and untreated severe patients, the “movement with cognition” (*p*‐value = 0.009) and “other MPS symptoms” (*p*‐value = 0.037) demonstrated a significant difference between groups using an ANOVA. The “movement” section had a *p*‐value of 0.055, and the “cognition” section had a *p*‐value of 0.53.

Attenuated ERT patients had a positive average in improvement for all sections except for “other MPS symptoms” (Table S1). Because there were no untreated attenuated patients with multiple surveys, no statistical significance could be determined.

Over time, “movement with cognition” and “cognition” scores had the largest declines in severe untreated patients (Figure 3). However, one outlier from the upper limit, patient number 18, as shown in purple, changed the correlation and trend of the linear regression when removed. “Movement with cognition” and “cognition” sections had no outliers. When excluding patient number 18, all scores declined over time (Figure 3).

**FIGURE 4:**
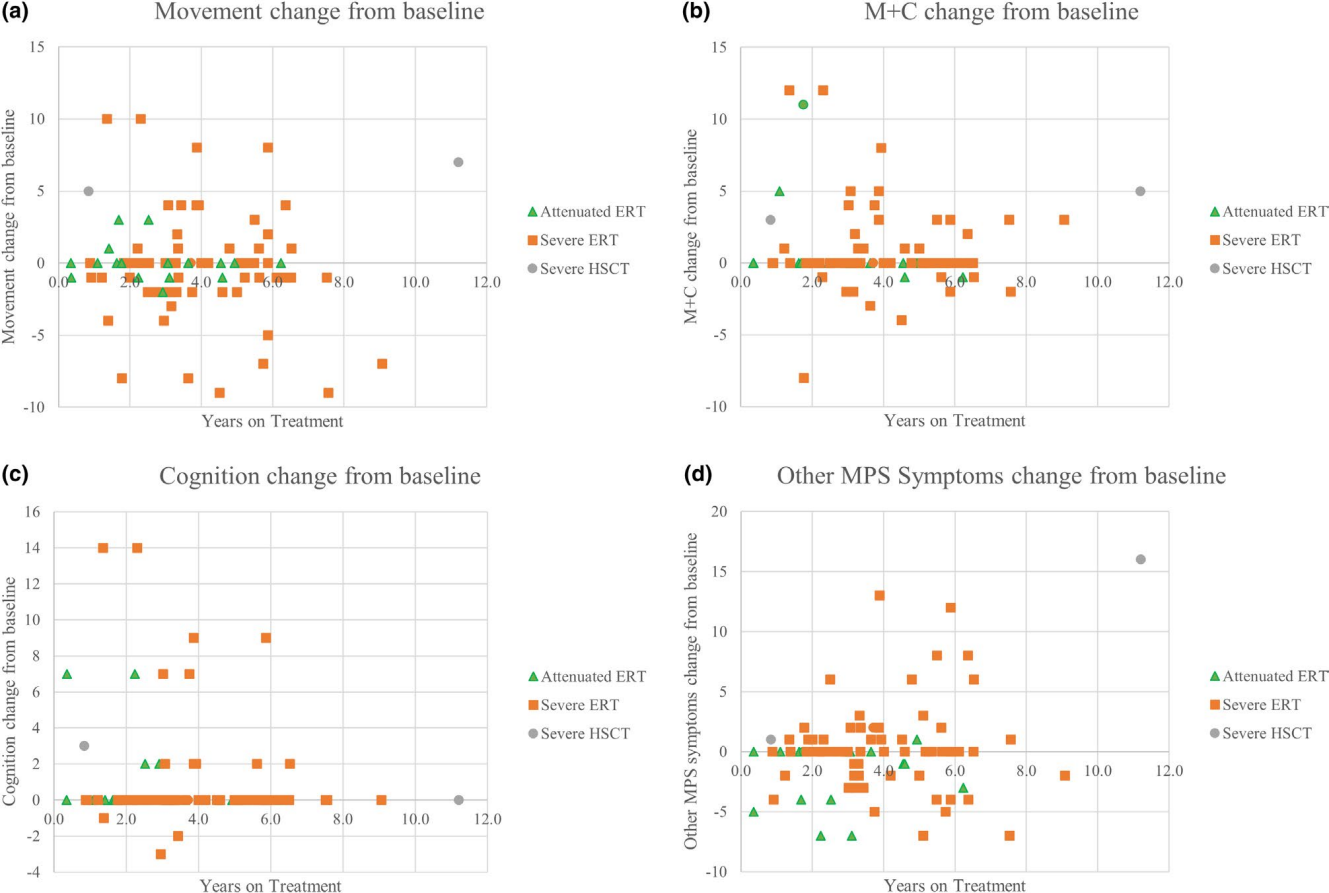
ADL score changes for treated patients. (a) “Movement” change in scores. Linear regression: *y* = −0.1352*x* + 0.3847. *R* ^2^ = 0.006. (b) “Movement with cognition” (M+C) change in scores. Linear regression: *y* = −0.0395*x* + 1.021. *R* ^2^ = 0.001. (c) “Cognition” change in scores. Linear regression: *y* = −0.1274*x* + 1.296. *R* ^2^ = 0.0085. (d) “Other MPS symptoms” change in scores. Linear regression: *y* = 0.486*x*–1.7768. *R* ^2^ = 0.062

The trendline for “movement,” “movement with cognition,” and “cognition” sections remained relatively flat, indicating the minimal change (Figure 4). The “other MPS symptoms” positive trendline indicated an increase over time on treatment (Figure 4).

### 3.8 Change in ADL scores by age

**FIGURE 5:**
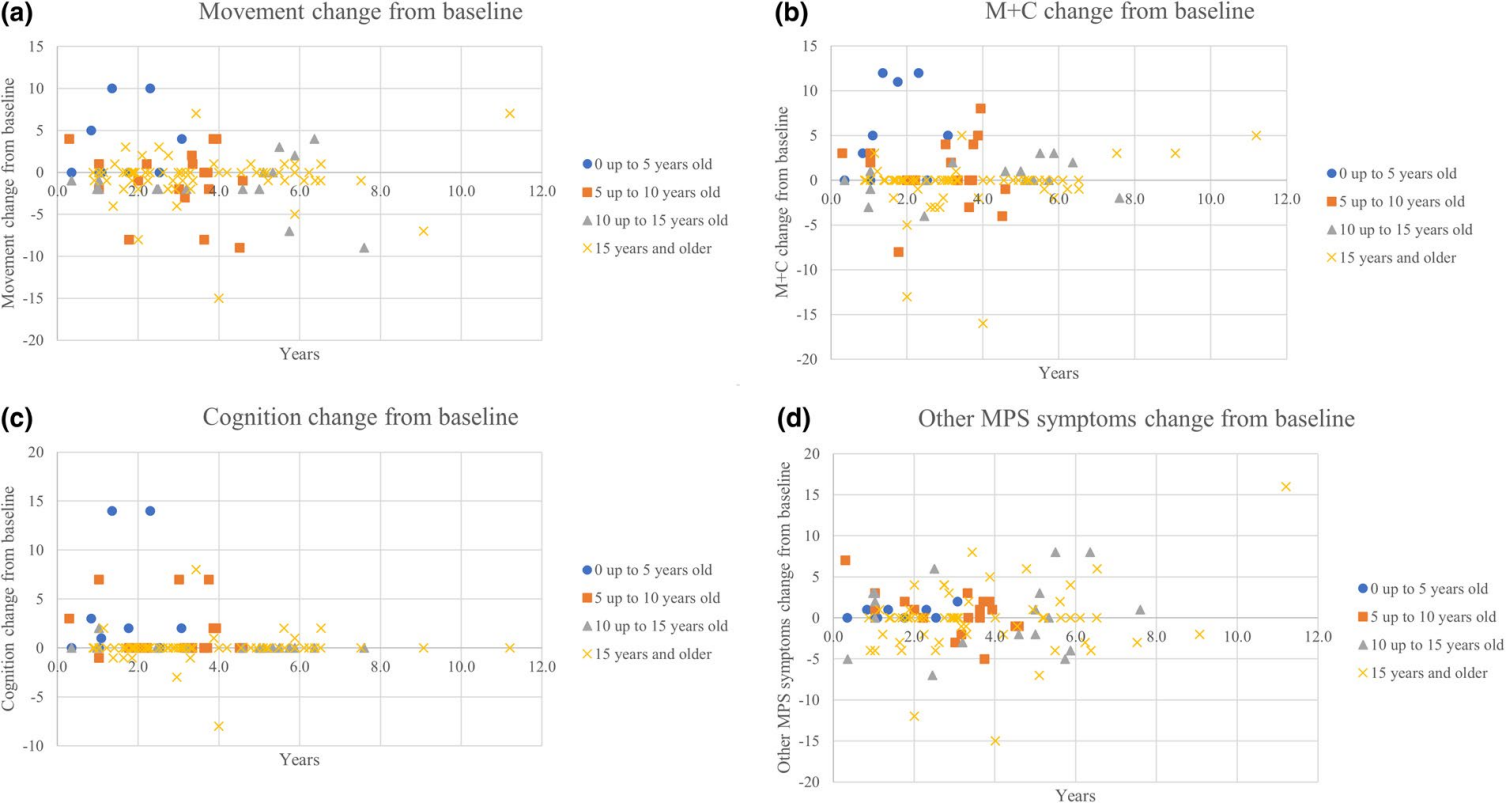
ADL score changes from baseline by age group. Untreated patients have the first survey as a baseline. Treated patients have the date of ERT initiation as a baseline. (a) “Movement” change in scores. (b) “Movement with cognition” (M+C) change in scores. (c) “Cognition” change in scores. (d) “Other MPS symptoms” change in scores

Because there were only 24 longitudinal surveys for untreated patients, all patients were included in each graph (Figure 5). The age group from 0 to 5 years had more significant changes in “movement” (3.22 ± 4.29) and “movement with cognition” (5.33 ± 5.15) scores compared to every other age group (*p*‐values for all age groups < 0.05). The age group of 5 to 10 years old had a more significant change in “cognition” (1.50 ± 2.71) than the age group 15 years and older (0.02 ± 1.58), with a *p*‐value of 0.04. Other score changes between age groups were not significant.

## 4 DISCUSSION

This study demonstrated that: (a) in MPS IVA patients, the “movement with cognition” and “cognition” scores increased with age, while “movement” and “other MPS symptoms” scores declined with age; (b) younger patients had higher changes in “movement,” “movement with cognition,” and “cognition” scores; (c) attenuated patients had higher scores in all categories at all ages compared to severe patients; (d) “cognition” scores in MPS IVA patients had the lowest variation and change after the age of 5 years; (e) severe ERT and HSCT patients still had lower scores compared to control (f) ERT and HSCT treatment improved only the “other MPS symptoms” section compared to untreated in severe patients; (g) ERT did not improve scores in any section compared to untreated in attenuated patients; (h) there were no differences between ERT and HSCT severe patient scores in any section; and (i) severe untreated patients generally decreased in scores with time in every section but physical training can improve “movement” and “other MPS symptoms.”

MPS IVA most severely affects the skeletal system, resulting in skeletal dysplasia and endurance limitations. The skeletal dysplasia leads to a more significant decrease in movement‐based sections and an indication for surgeries on the hip, legs, trachea, and spinal cord. However, “cognition” scores should not be affected in MPS IVA patients because the disorder does not affect cognitive development, unlike MPS I, II, III, and VII (Shapiro et al., 2017). Thus, the decreases in the “movement with cognition” section for MPS IVA patients have mainly attributed to “movement” section decreases. The decreasing trends in movement‐based scores match observations seen in previously published data (Yasuda et al., 2016). The “cognition” was the closest to age‐matched controls, as most patients reach the maximum score in “cognition” regardless of treatment. Some patients do not reach the maximum score regardless of treatment. This has been documented in a previous study where a minority of MPS IVA patients had a below‐average cognitive assessment (Davison et al., 2013). The patients over 20 years old who have “cognition” scores under 15 also have low “movement” scores (range 0–13). One explanation could be that because patients are restricted physically from movement functioning, they cannot successfully socialize in environments where they can learn social participation, conversation, problem‐solving, and understanding as evaluated in the survey. This theory was also proposed by Yasuda et al. (Yasuda et al., 2016).

Attenuated MPS IVA patients had lower “movement” scores than age‐matched controls but similar scores in “movement with cognition” and “cognition” scores. Severe MPS IVA patients had lower “movement,” “movement with cognition,” and “cognition” scores compared to age‐matched controls. Both observations are expected as the severe phenotype has earlier and more debilitating symptoms than the attenuated phenotype (Baujat & Valayannopoulos, 2014).

When determining the effects of treatment, average scores and changes in scores were evaluated. Regardless of phenotype or treatment, “other MPS symptoms” decreased with age. Severe ERT and HSCT patients had lower scores in all sections except for “cognition” for HSCT patients compared to control. This demonstrates that ERT has limitations in reversing skeletal effects, explaining these low scores in “movement” and “movement with cognition” compared to controls (Tomatsu et al., 2015). Attenuated ERT patients’ scores were equivalent to control because the averages were not significantly different (Table S1). However, when comparing with attenuated untreated patients, ERT patients did not differ in any section. This could be due to attenuated patients not having much of a deficit in treatment scores to improve.

ERT and HSCT increased only “other MPS symptoms” scores in severe patients, which differed from previously reported data (Cleary et al., 2021; Hendriksz et al., 2015, 2018; Moisan et al., 2020). Previous clinical trials with ERT showed an improvement in mobility using the health assessment questionnaire and endurance in the 6‐minute walk test, similar to the “movement” section in this study (Cleary et al., 2021; Hendriksz et al., 2015, 2018; Moisan et al., 2020). Severe ERT patients had a numerical increase in “movement” compared to severe untreated patients without statistical significance. Clinical trials have also reported no improvement in caregiver‐assistance and self‐care sections, which matches this study's data on the “movement with cognition” sections because both evaluate eating, bathing, and dressing independently (Hendriksz et al., 2015, 2018).

In contrast to ERT, HSCT is only administered once, and fewer patients receive this treatment. This study showed that severe patients with HSCT improved in “other MPS symptoms” compared to severe untreated patients. One patient also reported improvements in “other MPS symptoms” after 9+ years post‐HSCT (Chinen et al., 2014). Other published cases reported a better score in patients who received HSCT under 5 years old and more significant improvement in movement‐based sections than the “cognition” section because “cognition” was closer to healthy controls (Yabe et al., 2016). This study showed a numerical improvement in HSCT patients compared to untreated patients in those sections, but it was not significant. More data could have provided a more accurate average to determine true significance.

There were no differences between ERT and HSCT severe patient scores in any section. No previous studies have compared ERT and HSCT improvements in ADL for MPS IVA patients, but there are published data on MPS II patients (Tanjuakio et al., 2015). In previous studies, HSCT provided a higher ADL score than early and late ERT, but the difference for early ERT was not significant (Tanjuakio et al., 2015). This study also demonstrates a higher “movement” score for severe HSCT patients than severe ERT patients without statistical significance. Our study concurs with previous studies promoting early treatment because of increased improvements with younger patients (Tanjuakio et al., 2015; Yabe et al., 2016).

It is notable that when symptoms uncommon to MPS IVA in the “other MPS symptoms” (such as behavioral problems, skin, and hair) were omitted, the differences between treated and untreated groups become more evident in severe patients. The altered scores for severe patients were 35.18 ± 5.05 for ERT, 38.43 ± 3.41 for HSCT, and 33.02 ± 6.92 for untreated. The *p*‐value between ERT and untreated severe patients changed from 0.0324 to 0.0097. The *p*‐value between HSCT and untreated severe patients changed from 0.0174 to 0.0042. In both cases, the *p*‐value decreased, suggesting more significant differentiation. When comparing severe ERT and HSCT patients, the *p*‐value changed from 0.0772 to 0.0464. This finding has shown a significant difference between ERT and HSCT groups in severe patients in the “other MPS symptoms” when excluding uncommon symptoms. In attenuated patients, the altered scores for attenuated patients were 37.09 ± 5.40 for ERT and 39.32 ± 4.06 for untreated patients. There was only one attenuated HSCT patient, so that the new score was 36. The *p*‐value remained insignificant when comparing ERT and untreated attenuated patients (0.1465 changed to 0.135).

One important observation unique to this study is that the increase in “movement” and “other MPS symptoms” scores is likely due to physical training. Patient number 18 (purple) is an outlier in those two sections, which alters the slope of the best fit line from positive to negative in “movement” and “other MPS symptoms” because it is an outlier (Figure 3). Notably, the patient represented by patient number 18 was wheelchair‐bound and crawling with severe muscle atrophy until he started physical training daily, including bike and weight training with dumbbells one year ago, which increased his muscle mass and improved his quality of life significantly. Currently, he is no longer wheelchair‐bound and is independent of day‐to‐day activities.

Limitations include having a small sample size for attenuated and HSCT patients, which makes determining the effect of treatment and comparing treatments difficult.

## 5 CONCLUSIONS

Overall, this study has confirmed the reliability of an ADL questionnaire to distinguish between control and MPS IVA patients and severe and attenuated phenotypes. The ADL questionnaire also demonstrated the decreased variability in “cognition” compared to “movement,” which is consistent in MPS IVA development. Younger patients had a larger ability to change ADL scores, which should be noted when evaluating patients for longitudinal improvement. Early intervention is key to the slow progression of the disease. The ADL questionnaire can also assess treatment efficacy as the “movement” scores were higher in ERT and HSCT patients than untreated patients without significance. There is no statistical difference when comparing long‐term effects of HSCT and ERT, but more patients with HSCT should be evaluated to confirm. This study also demonstrates that physical training can significantly improve “movement” scores, despite lacking treatment. A clinical trial evaluating the effects of physical training on untreated patients could establish a recommended regimen to improve movement within limitations from skeletal dysplasia.

## CONFLICTS OF INTEREST

The authors declare no conflict of interest.

## INSTITUTIONAL REVIEW BOARD STATEMENT

The study was conducted according to the guidelines of the Declaration of Helsinki and approved by the Institutional Review Board of Nemours/AIDHC (IRB #750932: 06/05/2015, initial approval: IRB # 750932; 02/10/2021, Amendment/Modification approval).

## Supporting information

Table S1‐S2

## Data Availability

All relevant data are within the manuscript and its Supporting Information files.
