# Supplementary material for: Activity of daily living in mucopolysaccharidosis IVA patients: Evaluation of therapeutic efficacy
Source: Mol Genet Genomic Med. 2021 Oct 8;9(11):e1806. doi: 10.1002/mgg3.1806 (PMC8606213; doi:10.1002/mgg3.1806)
Supplement: Supplementary file 1 — Table S1‐S2 [file MGG3-9-e1806-s001.docx]

**Supplementary Table 1.** ADL scores in controls and patients with severe phenotypes or attenuated phenotypes.

|  |  | **Control** | **Severe** | | | **Attenuated** | | | |
| --- | --- | --- | --- | --- | --- | --- | --- | --- | --- |
|  |  |  | **ERT** | **HSCT** | **Untreated** | **ERT** | **HSCT** | **Untreated** | |
| **Number of surveys** |  | 145 | 113 | 7 | 105 | 23 | 1 | 19 |  |
|  | **Movement** | 18.18 ± 4.08 | 11.29 ± 4.60*** | 13.71 ± 4.68* | 10.51 ± 5.44*** | 14.57 ± 3.75 | 17 | 16.79 ± 2.30* | |
|  | **Movement with Cognition** | 16.41 ± 6.13 | 13.82 ± 3.94*** | 10.43 ± 5.32* | 12.90 ± 5.01*** | 17.83 ± 2.29 | 19 | 17.68 ± 3.42 | |
|  | **Cognition** | 14.79 ± 5.54 | 18.24 ± 3.14*** | 13.71 ± 7.18 | 17.42 ± 4.05*** | 19.61 ± 1.08 | 19 | 19.26 ± 1.63*** | |
|  | **Other MPS Symptoms** | - | 48.88 ± 5.79 | 52.86 ± 4.88 | 46.93 ± 7.42 | 51.39 ± 5.82 | 51 | 53.79 ± 4.67 | |
| **t-tests between treatment groups and untreated** | **Movement** |  | p = 0.2574 | p = 0.1260 | - | p = 0.0238 | - | - | |
|  | **Movement with Cognition** |  | p = 0.1363 | p = 0.2718 | - | p = 0.8782 | - | - | |
|  | **Cognition** |  | p = 0.0982 | p = 0.2236 | - | p = 0.4339 | - | - | |
|  | **Other MPS Symptoms** |  | p = 0.0324 | p = 0.0174 | - | p = 0.1465 | - | - | |
| **t-tests between ERT and HSCT** | **Movement** |  | p = 0.2268 |  |  |  |  |  | |
|  | **Movement with Cognition** |  | p = 0.1447 |  |  |  |  |  | |
|  | **Cognition** |  | p = 0.1474 |  |  |  |  |  | |
|  | **Other MPS Symptoms** |  | p = 0.0772 |  |  |  |  |  | |

Note;

t-test p-values:

*p < 0.05 of scores compared to control.

**p < 0.01 of scores compared to control.

***p < 0.001 of scores compared to control.

ANOVA p-values:

Control and severe groups ERT, HSCT, untreated: “movement,” “movement with cognition,” and “cognition” p-value < 0.001.

Severe groups ERT, HSCT, untreated: “movement” p-value = 0.18, “movement with cognition” p-value = 0.078, “cognition” p-value = 0.005, “other MPS symptoms” p-value = 0.015.

Control and attenuated groups ERT, HSCT, untreated: “movement” and “cognition” p-values < 0.001, “movement with cognition” p-value = 0.55.

Attenuated groups ERT, HSCT, untreated: “movement” p-value = 0.083, “movement with cognition,” “cognition,” “other MPS symptoms” p-value > 0.1.

**Supplementary Table 2.** ADL scores in controls and patients with severe phenotypes or attenuated phenotypes.

|  |  | **Severe** | | | **Attenuated** | | |
| --- | --- | --- | --- | --- | --- | --- | --- |
|  |  | **ERT** | **HSCT** | **Untreated** | **ERT** | **HSCT** | **Untreated** |
| **Number of surveys** |  | 65 | 2 | 24 | 18 | 0 | 0 |
|  | **Movement** | -0.37 ± 3.79 | 6 ± 1.41 | -0.96 ± 4.10 | 0.06 ± 1.26 | - | - |
|  | **Movement with Cognition** | 0.80 ± 2.98* | 4 ± 1.41 | -1.5 ± 4.67 | 0.78 ± 2.84 | - | - |
|  | **Cognition** | 0.97 ± 3.13 | 1.5 ± 2.12 | 0.21 ± 2.43 | 0.17 ± 0.51 | - | - |
|  | **Other MPS Symptoms** | 0.31 ± 3.67 | 8.5 ± 10.61 | 0.13 ± 5.65 | -1.72 ± 2.59 | - | - |

Note;

t-test p-values:

*p < 0.05 of treatment group scores compared to untreated.

ANOVA p-values:

Severe groups ERT, HSCT, untreated: “movement” p-value = 0.055, “movement with cognition” p-value = 0.009, “cognition” p-value = 0.53, “other MPS symptoms” p-value = 0.037.
